# X-ray structure analysis of symmetrically substituted 1,1′-diformyl­ruthenocene

**DOI:** 10.1107/S2056989018010642

**Published:** 2018-08-10

**Authors:** Bertin Anzaldo, Pankaj Sharma, Francisco Lara Ochoa, Claudia P. Villamizar C., René Gutiérrez Pérez

**Affiliations:** a Instituto de Química Universidad Autónoma de México UNAM, Cd., Universitaria, PO Box 04510, Ciudad de México, Mexico; b Lab. Síntesis de Complejos, Fac. Cs. Quím.-BUAP, Ciudad Universitaria, PO Box 72592 Puebla, Mexico

**Keywords:** ruthenocene, 1,1′-di­formyl­ruthenocene, X-ray structure, crystal structure

## Abstract

1,1′-Di­formyl­ruthenocene, [Ru(C_6_H_5_O)_2_], crystallizes in the ortho­rhom­bic system in the *P*2_1_2_1_2_1_ space group at room temperature. There are two crystallographically independent mol­ecules in the asymmetric unit. The cyclo­penta­dienyl rings have eclipsed configuration. The mol­ecules self-assemble in a two-dimensional structure by C—H⋯O and C—H⋯π inter­actions with *cisoid* relative orientations of the two formyl groups. The crystal studied was refined as an inversion twin.

## Abstract 2

The crystal and mol­ecular structure of 1,1′-formyl­ruthenocene has been determined. The mol­ecules self-assemble in a two-dimensional structure by C—H⋯O and C—H⋯π inter­actions with *cisoid* relative orientations of the two formyl groups.

## Chemical context   

Ferrocene and its derivatives are among the most important metallocenes. The general chemistry of ruthenocenes and osmocenes has not been researched much, as they are much less reactive and much more expensive. The ruthenocene skeleton is less ‘superaromatic’ than the ferrocene skeleton (Nesmeyanov *et al.*, 1972 ▸). It has long been reported that ruthenocene is more reactive towards li­thia­tion than its ferrocene analogue (Rausch *et al.*, 1960 ▸). The presence of less negative charge on the cyclo­penta­dienyl rings of ruthenocene than on those of ferrocene explains this higher reactivity as well as the higher acidity of the ruthenocene system (Sanders & Mueller-Westerhoff, 1996 ▸).

Numerous applications of 1,1′-disubstituted derivatives of ferrocene and ruthenocene in asymmetric catalysis (Dai & Hou, 2010 ▸), biochemistry and material sciences (Štěpnička, 2008 ▸), have been reported. Different types of substituents on the Cp ring often result in significant changes in the reactivity and properties of ruthenocene as a result of the electronic and steric factors that influence the mol­ecular entity. In general, ferrocene (Fc) and its heavier analogue ruthenocene (Rc) have similar structures (Muratov *et al.*, 2014 ▸). The mol­ecular structures of formyl ferrocene, 1,1′-diformyl ferrocene and formyl ruthenocene are known in the literature (Braga *et al.*, 1999 ▸; Muratov *et al.*, 2014 ▸). The structures of 1,1′-disubstituted ferrocenes containing carb­oxy­lic or carbonyl groups have the potential to form a large number of inter­molecular inter­actions, building blocks in two or three dimensions, and to mould the inter­molecular hydrogen bonds and CO networks to achieve highly organized superstructures (Braga & Grepioni, 1997 ▸). The structure of the ferrocene analogue of the title compound has been published (Braga *et al.*, 1999 ▸; MacGillivray *et al.*, 1999 ▸). We report here the crystal and mol­ecular structure of 1,1′-diformyl­ruthenocene, which has not previously been reported.

## Structural commentary   

**Figure 1:**
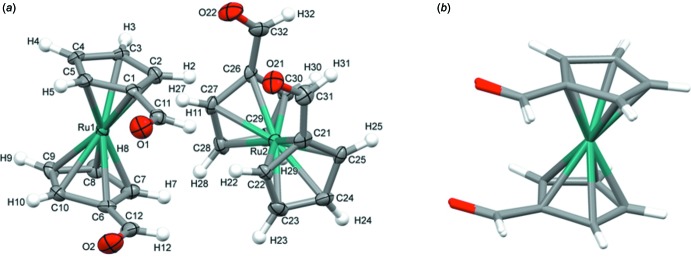
(*a*) *ORTEP* representation of the two crystallographically independent ruthenocene complex mol­ecules in the asymmetric unit at 50% probability level with atomic labelling. (*b*) The superimposed mol­ecules from the asymmetric unit.

The title compound contains two crystallographic independent mol­ecules (*A* and *B*, Fig. 1 ▸ *a*) in the asymmetric unit, which possess the same rotameric conformations. In both mol­ecules, the carbon atoms of the cyclo­penta­dienyl rings form penta­gonal prisms, which bind to the ruthenium atom (sandwich array). These Cp rings are in partially eclipsed positions. The two –CHO groups of the cyclo­penta­dienyl rings are in *cisoid* relative conformations. Bond lengths in the two independent mol­ecules are given in Table 1 ▸. The C1—C11—C12—C6 and C31—C21—C26—C32 torsion angles are 2.5 (9) and 6.0 (9)°, respectively, which suggests that mol­ecule *A* is more eclipsed than mol­ecule *B*. In the reported crystal structure of the Fc(CHO)_2_ analogue, there are also two independent mol­ecular units in the asymmetric unit, but with different rotameric conformations. Similarly, the torsion angles C11—O1—O2—C12 and C31—O21—O22—C32 are 2 (1) and 7 (1)°, respectively. The torsion angle in diformyl ferrocene, which has a staggered configuration in one of the mol­ecules in the asymmetric unit is 42.4° (Balavoine *et al.*, 1991 ▸; Mueller-Westerhoff *et al.*, 1993 ▸). The crystal structure of the di­acetyl­ruthenocene mol­ecule reported earlier also shows a *cis* configuration for the acetyl group, but one acetyl group is rotated by 180° with respect to the other (Trotter, 1963 ▸).
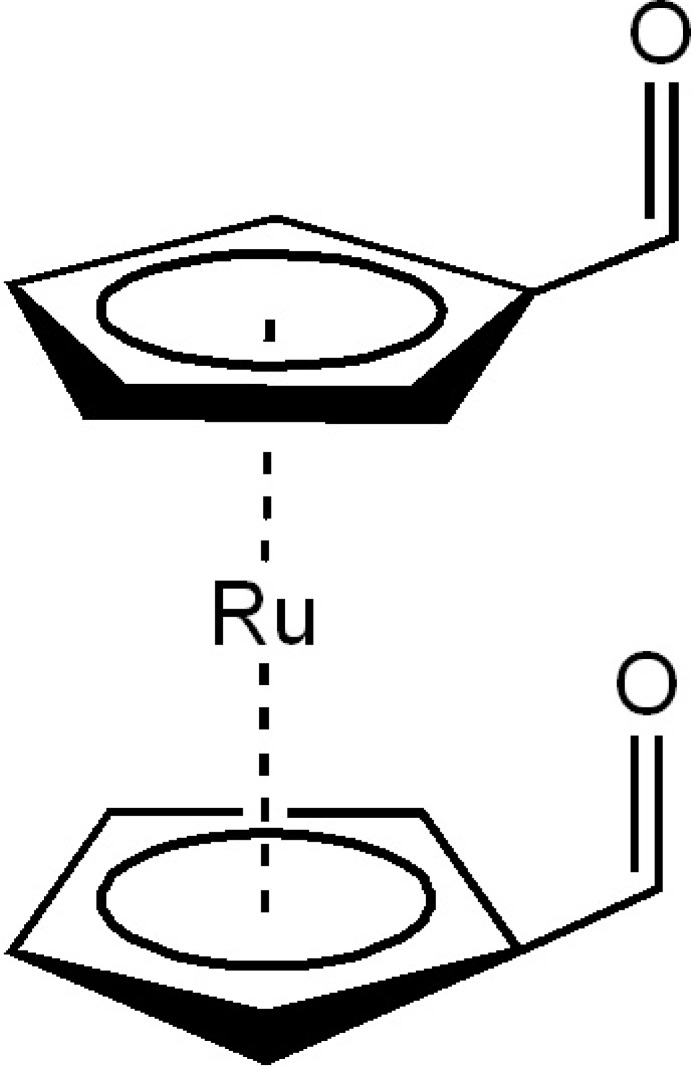


The Cp(centroid)⋯Cp(centroid) distances in mol­ecules *A* and *B* are 3.621 and 3.616 Å, respectively. The difference could be due to the electronic effects of the two symmetrically substituted formyl groups. It was also observed that the C and O atoms of both formyl groups are nearly coplanar to the plane of their respective Cp ring. A comparison of the two complex mol­ecules in the asymmetric unit was performed by calculation of the mol­ecular overlay (*Mercury*; Macrae *et al.*, 2008 ▸) (Fig. 1 ▸ *b*), resulting in the values *D*_r.m.s._ = 0.0622 and *D*_max_ = 0.1208.

## Supra­molecular features   

**Figure 2:**
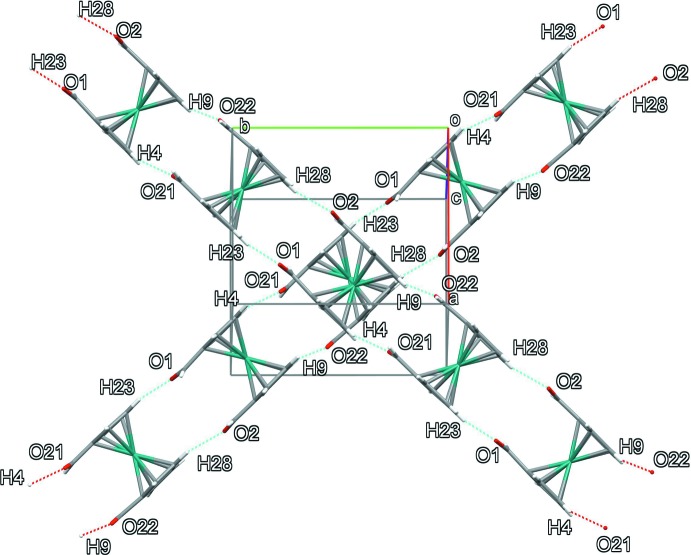
Packing arrangement showing the perpendicular columns formed by the C= O⋯H—Cp secondary inter­actions.

The mol­ecules self-assemble in a two-dimensional structure assisted by C—H⋯O and C—H⋯π inter­actions (Desiraju, 1996 ▸), as shown in Fig. 2 ▸. Numerical details are given in Table 2 ▸. All secondary inter­actions that are shorter than the sum of the van der Waals radii of the atoms involved minus 0.12 Å are included. The mol­ecules form columns that are arranged in two-dimensional sheets parallel to the *ab* plane.

**Figure 3:**
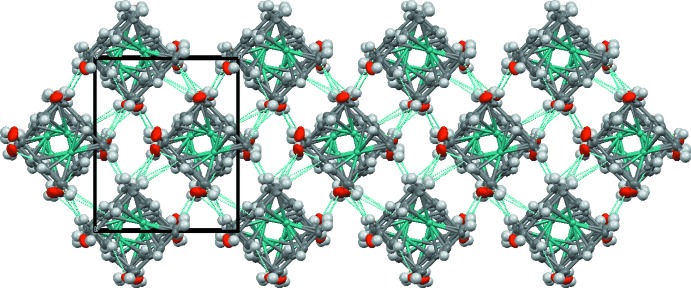
Packing arrangement along the c axis with dashed lines indicating the inter­molecular contacts.

The short contacts of each mol­ecule result in a special neighbouring array in three-dimensions, forming V-type assemblies as shown in Fig. 3 ▸. In particular, the space group *P*2_1_2_1_2_1_ permits close packing of mol­ecules (Braga *et al.*, 1999 ▸).

## Quantum-chemical calculations   

DFT quantum-chemical calculations were performed using ωB97X-D based on 6-31 G* with *SPARTAN16* (Wavefunction, 2017 ▸). The DFT structure optimization of 1,1′-diformyl­ruthenocene was performed starting from the X-ray data. The energy of mol­ecule *A*, where the mol­ecule is eclipsed and the formyl groups are in a *cisoid* geometry, is 0.73 Kcal more stable than that of the mol­ecule with a *transoid* geometry for the two formyl groups. When the energy of the two mol­ecules calculated together was compared with the sum of the energies obtained independently for each mol­ecule, it was observed that the asymmetric unit *A*–*B* is more stable by 14.14 Kcal. This observation may be partly due to the presence of the two C—H⋯O hydrogen-bonding inter­actions between the two independent mol­ecules as shown in Fig. 3 ▸.

## Synthesis and crystallization   

All reactants were purchased from Aldrich Chemical Co. and 1,1′-diformyl ruthenocene was synthesized as reported earlier (Trotter, 1963 ▸). Yellow needle-like crystals of ruthenocene di­aldehyde were obtained by slow evaporation of a saturated di­chloro­methane/hexane solution (*v*:*v* = 2:8) at ambient temperature.

## Refinement   

Crystal data, data collection and structure refinement details are summarized in Table 3 ▸. H atoms were positioned geometrically and refined using a riding model: C—H = 0.93–0.98 Å with *U*_iso_(H) = 1.2*U*_eq_(C).

## Supplementary Material

Crystal structure: contains datablock(s) I. DOI: 10.1107/S2056989018010642/zp2025sup1.cif

Structure factors: contains datablock(s) I. DOI: 10.1107/S2056989018010642/zp2025Isup2.hkl

CCDC reference: 1545792

Additional supporting information:  crystallographic information; 3D view; checkCIF report

## Figures and Tables

**Table 1:** Lengths of the Ru—C and C—C bonds in the Cp rings of the *A* and *B* mol­ecules.

*A*		*B*	
Ru1—C1	2.175 (10)	Ru2—C21	2.154 (10)
Ru1—C2	2.173 (11)	Ru2—C22	2.166 (10)
Ru1—C3	2.198 (11)	Ru2—C23	2.182 (12)
Ru1—C4	2.190 (11)	Ru2—C24	2.197 (12)
Ru1—C5	2.166 (11)	Ru2—C25	2.177 (11)
Ru1—C6	2.153 (10)	Ru2—C26	2.160 (11)
Ru1—C7	2.161 (12)	Ru2—C27	2.169 (11)
Ru1—C8	2.175 (12)	Ru2—C28	2.172 (12)
Ru1—C9	2.190 (12)	Ru2—C29	2.179 (11)
Ru1—C10	2.173 (11)	Ru2—C30	2.177 (11)
C1—C2	1.430 (15)	C21—C25	1.430 (16)
C2—C3	1.385 (15)	C21—C22	1.407 (15)
C3—C4	1.425 (15)	C22—C23	1.408 (17)
C4—C5	1.403 (16)	C23—C24	1.405 (17)
C1—C5	1.418 (15)	C24—C25	1.415 (17)
C6—C7	1.425 (16)	C26—C30	1.449 (15)
C7—C8	1.416 (17)	C26—C27	1.410 (15)
C8—C9	1.448 (16)	C27—C28	1.410 (17)
C9—C10	1.389 (17)	C28—C29	1.431 (16)
C6—C10	1.425 (16)	C29—C30	1.402 (16)

**Table 2:** Hydrogen-bond geometry (Å, °) *Cg* is the centroid of the C1–C5 ring.

*D*—H⋯*A*	*D*—H	H⋯*A*	*D*⋯*A*	*D*—H⋯*A*
C2—H2⋯O21	0.98	2.64	3.456 (16)	141
C3—H3⋯O1^i^	0.98	2.62	3.536 (15)	156
C4—H4⋯O21^i^	0.98	2.45	3.406 (16)	164
C9—H9⋯O22^i^	0.98	2.42	3.370 (16)	162
C23—H23⋯O1^ii^	0.98	2.44	3.379 (17)	161
C28—H28⋯O2^ii^	0.98	2.43	3.392 (16)	167
C30—H30⋯O2^iii^	0.98	2.63	3.436 (14)	140
C8—H8⋯*Cg* ^i^	0.98	2.78	3.546 (15)	135

Symmetry codes: (i) 
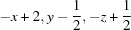
; (ii) 
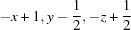
; (iii) 
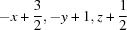
.

**Table 3:** Experimental details

Crystal data	
Chemical formula	[Ru(C_6_H_5_O)_2_]
*M*_r_	287.27
Crystal system, space group	Orthorhombic, *P*2_1_2_1_2_1_
Temperature (K)	298
*a*, *b*, *c* (Å)	8.944 (2), 10.797 (3), 20.520 (5)
*V* (Å^3^)	1981.6 (8)
*Z*	8
Radiation type	Mo *K*α
μ (mm^−1^)	1.55
Crystal size (mm)	0.48 × 0.12 × 0.05

Data collection	
Diffractometer	Bruker SMART APEX CCD
Absorption correction	Multi-scan (*SADABS*; Bruker, 2012 ▸)
*T*_min_, *T*_max_	0.499, 0.922
No. of measured, independent and observed [*I* > 2σ(*I*)] reflections	6151, 4288, 3556
*R*_int_	0.048
(sin θ/λ)_max_ (Å^−1^)	0.641

Refinement	
*R*[*F* ^2^ > 2σ(*F* ^2^)], *wR*(*F* ^2^), *S*	0.053, 0.123, 1.03
No. of reflections	4288
No. of parameters	272
H-atom treatment	H-atom parameters constrained
Δρ_max_, Δρ_min_ (e Å^−3^)	1.28, −0.68
Absolute structure	Refined as an inversion twin
Absolute structure parameter	0.43 (12)

Computer programs: *APEX2* and *SAINT* (Bruker, 2012 ▸), *XP* in *SHELXTL* and *SHELXS2012* (Sheldrick, 2008 ▸), *SHELXL2014* (Sheldrick, 2015 ▸) and *CIFTAB* (Sheldrick, 2013).

## supplementary crystallographic information

### Crystal data

**Table:**

[Ru(C_6_H_5_O)_2_]	*D*_x_ = 1.926 Mg m^−^^3^
*M**_r_* = 287.27	Mo *K*α radiation, λ = 0.71073 Å
Orthorhombic, *P*2_1_2_1_2_1_	Cell parameters from 3163 reflections
*a* = 8.944 (2) Å	θ = 2.5–27.4°
*b* = 10.797 (3) Å	µ = 1.55 mm^−^^1^
*c* = 20.520 (5) Å	*T* = 298 K
*V* = 1981.6 (8) Å^3^	Needle, yellow
*Z* = 8	0.48 × 0.12 × 0.05 mm
*F*(000) = 1136	

### Data collection

**Table:**

Bruker SMART APEX CCD diffractometer	4288 independent reflections
Radiation source: fine-focus sealed tube	3556 reflections with *I* > 2σ(*I*)
Graphite monochromator	*R*_int_ = 0.048
Detector resolution: 8.333 pixels mm^-1^	θ_max_ = 27.1°, θ_min_ = 2.0°
ω scans	*h* = −7→11
Absorption correction: multi-scan (SADABS; Bruker, 2012)	*k* = −13→8
*T*_min_ = 0.499, *T*_max_ = 0.922	*l* = −26→26
6151 measured reflections	

### Refinement

**Table:**

Refinement on *F*^2^	Secondary atom site location: difference Fourier map
Least-squares matrix: full	Hydrogen site location: inferred from neighbouring sites
*R*[*F*^2^ > 2σ(*F*^2^)] = 0.053	H-atom parameters constrained
*wR*(*F*^2^) = 0.123	*w* = 1/[σ^2^(*F*_o_^2^) + (0.0369*P*)^2^] where *P* = (*F*_o_^2^ + 2*F*_c_^2^)/3
*S* = 1.02	(Δ/σ)_max_ < 0.001
4288 reflections	Δρ_max_ = 1.28 e Å^−^^3^
272 parameters	Δρ_min_ = −0.68 e Å^−^^3^
0 restraints	Absolute structure: Refined as an inversion twin
Primary atom site location: structure-invariant direct methods	Absolute structure parameter: 0.43 (12)

### Special details

**Table:**

Geometry. All esds (except the esd in the dihedral angle between two l.s. planes) are estimated using the full covariance matrix. The cell esds are taken into account individually in the estimation of esds in distances, angles and torsion angles; correlations between esds in cell parameters are only used when they are defined by crystal symmetry. An approximate (isotropic) treatment of cell esds is used for estimating esds involving l.s. planes.
Refinement. Refined as a two-component inversion twin

### Fractional atomic coordinates and isotropic or equivalent isotropic displacement parameters (Å^2^)

**Table:**

	*x*	*y*	*z*	*U*_iso_*/*U*_eq_	
Ru1	0.80791 (9)	0.43584 (8)	0.21733 (4)	0.0277 (2)	
O1	0.7067 (12)	0.7801 (9)	0.1729 (5)	0.061 (3)	
O2	0.4211 (10)	0.5356 (10)	0.1480 (5)	0.065 (3)	
C1	0.8625 (12)	0.6319 (9)	0.2228 (6)	0.033 (3)	
C2	0.9145 (12)	0.5713 (11)	0.2804 (6)	0.037 (3)	
H2	0.8837	0.5912	0.3250	0.045*	
C3	1.0219 (12)	0.4849 (11)	0.2632 (6)	0.036 (3)	
H3	1.0794	0.4330	0.2933	0.043*	
C4	1.0382 (12)	0.4888 (11)	0.1942 (6)	0.038 (3)	
H4	1.1091	0.4397	0.1685	0.045*	
C5	0.9390 (12)	0.5777 (12)	0.1693 (5)	0.038 (3)	
H5	0.9302	0.6028	0.1236	0.046*	
C6	0.5752 (11)	0.3939 (11)	0.2021 (6)	0.034 (3)	
C7	0.6230 (13)	0.3378 (11)	0.2614 (6)	0.039 (3)	
H7	0.5812	0.3536	0.3047	0.047*	
C8	0.7370 (15)	0.2513 (12)	0.2465 (6)	0.045 (3)	
H8	0.7882	0.1966	0.2774	0.054*	
C9	0.7569 (13)	0.2533 (11)	0.1764 (6)	0.038 (3)	
H9	0.8262	0.2011	0.1516	0.045*	
C10	0.6593 (14)	0.3402 (12)	0.1503 (6)	0.042 (3)	
H10	0.6475	0.3589	0.1039	0.050*	
C11	0.7431 (12)	0.7226 (10)	0.2206 (6)	0.038 (3)	
H11	0.6906	0.7374	0.2589	0.046*	
C12	0.4648 (13)	0.4892 (13)	0.1974 (6)	0.047 (3)	
H12	0.4233	0.5177	0.2361	0.057*	
Ru2	0.66487 (9)	0.45422 (8)	0.46810 (4)	0.0282 (2)	
O21	0.7700 (12)	0.7744 (9)	0.3943 (5)	0.062 (3)	
O22	1.0724 (10)	0.5577 (11)	0.4277 (5)	0.064 (3)	
C21	0.6048 (13)	0.6452 (10)	0.4527 (6)	0.036 (3)	
C22	0.5427 (12)	0.5738 (10)	0.4021 (5)	0.031 (2)	
H22	0.5617	0.5840	0.3554	0.038*	
C23	0.4408 (14)	0.4905 (12)	0.4307 (7)	0.046 (3)	
H23	0.3775	0.4313	0.4072	0.055*	
C24	0.4395 (13)	0.5115 (12)	0.4982 (7)	0.045 (3)	
H24	0.3765	0.4677	0.5298	0.054*	
C25	0.5434 (13)	0.6059 (11)	0.5137 (5)	0.036 (3)	
H25	0.5622	0.6423	0.5567	0.043*	
C26	0.9013 (12)	0.4147 (9)	0.4733 (5)	0.033 (2)	
C27	0.8409 (14)	0.3525 (11)	0.4189 (6)	0.037 (3)	
H27	0.8719	0.3641	0.3735	0.045*	
C28	0.7321 (14)	0.2679 (12)	0.4410 (6)	0.042 (3)	
H28	0.6749	0.2100	0.4140	0.051*	
C29	0.7277 (14)	0.2767 (11)	0.5106 (6)	0.042 (3)	
H29	0.6644	0.2264	0.5392	0.051*	
C30	0.8295 (12)	0.3670 (10)	0.5314 (5)	0.035 (2)	
H30	0.8524	0.3897	0.5765	0.042*	
C31	0.7200 (15)	0.7386 (11)	0.4468 (7)	0.048 (3)	
H31	0.7581	0.7735	0.4848	0.057*	
C32	1.0095 (12)	0.5178 (11)	0.4742 (7)	0.044 (3)	
H32	1.0301	0.5543	0.5142	0.053*	

### Atomic displacement parameters (Å^2^)

**Table:**

	*U*^11^	*U*^22^	*U*^33^	*U*^12^	*U*^13^	*U*^23^
Ru1	0.0257 (4)	0.0277 (4)	0.0298 (4)	0.0009 (4)	0.0001 (4)	−0.0011 (4)
O1	0.070 (7)	0.052 (6)	0.061 (6)	0.010 (6)	−0.008 (6)	0.012 (5)
O2	0.050 (5)	0.075 (7)	0.071 (6)	0.017 (6)	−0.012 (5)	0.023 (6)
C1	0.032 (6)	0.023 (5)	0.044 (7)	−0.005 (4)	−0.002 (5)	−0.006 (5)
C2	0.042 (6)	0.037 (6)	0.033 (6)	0.004 (5)	−0.006 (5)	−0.004 (6)
C3	0.021 (5)	0.038 (7)	0.048 (7)	0.003 (5)	−0.002 (5)	0.002 (5)
C4	0.020 (5)	0.049 (7)	0.044 (6)	−0.001 (5)	0.006 (5)	−0.001 (6)
C5	0.030 (6)	0.051 (8)	0.034 (6)	−0.013 (6)	0.004 (5)	0.000 (6)
C6	0.023 (5)	0.032 (6)	0.046 (7)	−0.003 (5)	−0.006 (5)	0.003 (5)
C7	0.033 (6)	0.035 (6)	0.050 (7)	0.001 (5)	−0.003 (5)	0.005 (6)
C8	0.054 (8)	0.032 (7)	0.049 (8)	−0.003 (6)	0.001 (6)	0.015 (6)
C9	0.046 (7)	0.021 (6)	0.046 (7)	0.003 (5)	−0.001 (6)	−0.008 (5)
C10	0.038 (7)	0.045 (7)	0.042 (7)	−0.006 (6)	−0.005 (6)	−0.012 (6)
C11	0.039 (6)	0.032 (6)	0.045 (7)	−0.002 (5)	−0.005 (6)	−0.007 (6)
C12	0.030 (6)	0.066 (9)	0.046 (7)	−0.006 (6)	0.001 (5)	0.003 (7)
Ru2	0.0265 (4)	0.0270 (4)	0.0310 (4)	−0.0004 (4)	−0.0024 (4)	−0.0014 (4)
O21	0.078 (8)	0.049 (6)	0.060 (6)	−0.014 (5)	0.018 (5)	0.010 (5)
O22	0.049 (5)	0.082 (7)	0.061 (6)	−0.015 (6)	0.008 (4)	0.028 (6)
C21	0.034 (6)	0.020 (5)	0.055 (8)	0.014 (5)	0.000 (6)	0.003 (5)
C22	0.035 (5)	0.026 (6)	0.033 (5)	0.006 (5)	−0.010 (5)	−0.003 (5)
C23	0.032 (7)	0.043 (8)	0.062 (8)	0.010 (6)	−0.017 (6)	−0.011 (6)
C24	0.025 (6)	0.045 (8)	0.065 (8)	−0.001 (5)	0.003 (6)	−0.003 (6)
C25	0.033 (6)	0.039 (7)	0.034 (6)	0.011 (5)	0.007 (5)	−0.001 (5)
C26	0.039 (6)	0.023 (5)	0.036 (6)	0.013 (5)	−0.002 (5)	−0.007 (5)
C27	0.038 (6)	0.040 (6)	0.034 (6)	0.015 (6)	0.006 (5)	−0.005 (5)
C28	0.050 (8)	0.037 (7)	0.040 (7)	0.009 (6)	−0.007 (6)	−0.012 (6)
C29	0.041 (7)	0.029 (6)	0.056 (8)	0.007 (5)	0.000 (6)	0.015 (6)
C30	0.030 (5)	0.047 (6)	0.028 (5)	−0.001 (5)	−0.004 (5)	0.005 (5)
C31	0.052 (8)	0.024 (6)	0.067 (9)	0.007 (6)	0.007 (7)	0.001 (6)
C32	0.027 (5)	0.048 (7)	0.058 (8)	0.001 (5)	−0.006 (6)	0.001 (7)

### Geometric parameters (Å, º)

**Table:**

Ru1—C6	2.153 (10)	Ru2—C21	2.154 (10)
Ru1—C7	2.161 (12)	Ru2—C26	2.160 (11)
Ru1—C5	2.166 (11)	Ru2—C22	2.166 (10)
Ru1—C2	2.173 (11)	Ru2—C27	2.169 (11)
Ru1—C10	2.173 (11)	Ru2—C28	2.172 (12)
Ru1—C8	2.175 (12)	Ru2—C25	2.177 (11)
Ru1—C1	2.175 (10)	Ru2—C30	2.177 (11)
Ru1—C4	2.190 (11)	Ru2—C29	2.179 (11)
Ru1—C9	2.190 (12)	Ru2—C23	2.182 (12)
Ru1—C3	2.198 (11)	Ru2—C24	2.197 (12)
O1—C11	1.203 (14)	O21—C31	1.229 (15)
O2—C12	1.196 (14)	O22—C32	1.188 (14)
C1—C5	1.418 (15)	C21—C22	1.407 (15)
C1—C2	1.430 (15)	C21—C25	1.430 (16)
C1—C11	1.450 (15)	C21—C31	1.447 (17)
C2—C3	1.385 (15)	C22—C23	1.408 (17)
C2—H2	0.9800	C22—H22	0.9800
C3—C4	1.425 (15)	C23—C24	1.405 (17)
C3—H3	0.9800	C23—H23	0.9800
C4—C5	1.403 (16)	C24—C25	1.415 (17)
C4—H4	0.9800	C24—H24	0.9800
C5—H5	0.9800	C25—H25	0.9800
C6—C7	1.425 (16)	C26—C27	1.410 (15)
C6—C10	1.425 (16)	C26—C30	1.449 (15)
C6—C12	1.430 (17)	C26—C32	1.475 (16)
C7—C8	1.416 (17)	C27—C28	1.410 (17)
C7—H7	0.9800	C27—H27	0.9800
C8—C9	1.448 (16)	C28—C29	1.431 (16)
C8—H8	0.9800	C28—H28	0.9800
C9—C10	1.389 (17)	C29—C30	1.402 (16)
C9—H9	0.9800	C29—H29	0.9800
C10—H10	0.9800	C30—H30	0.9800
C11—H11	0.9300	C31—H31	0.9300
C12—H12	0.9300	C32—H32	0.9300
			
C6—Ru1—C7	38.6 (4)	C21—Ru2—C26	116.1 (4)
C6—Ru1—C5	127.3 (4)	C21—Ru2—C22	38.0 (4)
C7—Ru1—C5	161.9 (4)	C26—Ru2—C22	130.0 (4)
C6—Ru1—C2	130.7 (4)	C21—Ru2—C27	126.7 (5)
C7—Ru1—C2	114.6 (5)	C26—Ru2—C27	38.0 (4)
C5—Ru1—C2	63.8 (4)	C22—Ru2—C27	112.1 (4)
C6—Ru1—C10	38.5 (4)	C21—Ru2—C28	156.6 (5)
C7—Ru1—C10	64.2 (5)	C26—Ru2—C28	63.8 (4)
C5—Ru1—C10	112.3 (4)	C22—Ru2—C28	122.1 (4)
C2—Ru1—C10	164.8 (4)	C27—Ru2—C28	37.9 (5)
C6—Ru1—C8	64.2 (5)	C21—Ru2—C25	38.6 (4)
C7—Ru1—C8	38.1 (5)	C26—Ru2—C25	128.0 (4)
C5—Ru1—C8	158.5 (5)	C22—Ru2—C25	64.4 (4)
C2—Ru1—C8	125.5 (5)	C27—Ru2—C25	160.6 (4)
C10—Ru1—C8	63.9 (5)	C28—Ru2—C25	160.9 (5)
C6—Ru1—C1	115.4 (4)	C21—Ru2—C30	132.1 (4)
C7—Ru1—C1	128.8 (5)	C26—Ru2—C30	39.0 (4)
C5—Ru1—C1	38.1 (4)	C22—Ru2—C30	166.3 (4)
C2—Ru1—C1	38.4 (4)	C27—Ru2—C30	64.4 (4)
C10—Ru1—C1	129.2 (5)	C28—Ru2—C30	64.2 (4)
C8—Ru1—C1	160.6 (5)	C25—Ru2—C30	114.0 (4)
C6—Ru1—C4	158.9 (4)	C21—Ru2—C29	164.8 (5)
C7—Ru1—C4	159.8 (4)	C26—Ru2—C29	63.5 (4)
C5—Ru1—C4	37.6 (4)	C22—Ru2—C29	154.7 (4)
C2—Ru1—C4	62.7 (4)	C27—Ru2—C29	63.5 (5)
C10—Ru1—C4	124.2 (5)	C28—Ru2—C29	38.4 (4)
C8—Ru1—C4	125.0 (5)	C25—Ru2—C29	128.2 (5)
C1—Ru1—C4	63.0 (4)	C30—Ru2—C29	37.5 (4)
C6—Ru1—C9	63.5 (4)	C21—Ru2—C23	63.1 (5)
C7—Ru1—C9	63.9 (5)	C26—Ru2—C23	162.2 (5)
C5—Ru1—C9	125.1 (5)	C22—Ru2—C23	37.8 (5)
C2—Ru1—C9	157.7 (4)	C27—Ru2—C23	126.4 (5)
C10—Ru1—C9	37.1 (4)	C28—Ru2—C23	109.3 (5)
C8—Ru1—C9	38.7 (4)	C25—Ru2—C23	63.7 (5)
C1—Ru1—C9	160.4 (4)	C30—Ru2—C23	155.3 (5)
C4—Ru1—C9	110.3 (5)	C29—Ru2—C23	122.4 (5)
C6—Ru1—C3	162.7 (4)	C21—Ru2—C24	62.8 (5)
C7—Ru1—C3	127.2 (4)	C26—Ru2—C24	160.0 (5)
C5—Ru1—C3	63.5 (4)	C22—Ru2—C24	63.0 (5)
C2—Ru1—C3	36.9 (4)	C27—Ru2—C24	160.0 (5)
C10—Ru1—C3	156.7 (4)	C28—Ru2—C24	125.9 (5)
C8—Ru1—C3	110.9 (5)	C25—Ru2—C24	37.7 (4)
C1—Ru1—C3	63.1 (4)	C30—Ru2—C24	125.1 (5)
C4—Ru1—C3	37.9 (4)	C29—Ru2—C24	111.8 (5)
C9—Ru1—C3	124.2 (4)	C23—Ru2—C24	37.4 (5)
C5—C1—C2	107.1 (10)	C22—C21—C25	109.3 (10)
C5—C1—C11	127.6 (12)	C22—C21—C31	127.0 (12)
C2—C1—C11	125.0 (11)	C25—C21—C31	123.6 (12)
C5—C1—Ru1	70.6 (6)	C22—C21—Ru2	71.5 (6)
C2—C1—Ru1	70.7 (6)	C25—C21—Ru2	71.6 (6)
C11—C1—Ru1	119.3 (7)	C31—C21—Ru2	120.1 (8)
C3—C2—C1	108.8 (10)	C21—C22—C23	107.4 (10)
C3—C2—Ru1	72.5 (7)	C21—C22—Ru2	70.5 (6)
C1—C2—Ru1	70.9 (6)	C23—C22—Ru2	71.7 (7)
C3—C2—H2	125.5	C21—C22—H22	126.3
C1—C2—H2	125.5	C23—C22—H22	126.3
Ru1—C2—H2	125.5	Ru2—C22—H22	126.3
C2—C3—C4	107.7 (10)	C24—C23—C22	108.2 (11)
C2—C3—Ru1	70.6 (6)	C24—C23—Ru2	71.9 (7)
C4—C3—Ru1	70.7 (6)	C22—C23—Ru2	70.5 (7)
C2—C3—H3	126.1	C24—C23—H23	125.9
C4—C3—H3	126.1	C22—C23—H23	125.9
Ru1—C3—H3	126.1	Ru2—C23—H23	125.9
C5—C4—C3	108.5 (10)	C23—C24—C25	109.4 (12)
C5—C4—Ru1	70.3 (6)	C23—C24—Ru2	70.7 (7)
C3—C4—Ru1	71.4 (6)	C25—C24—Ru2	70.3 (6)
C5—C4—H4	125.8	C23—C24—H24	125.3
C3—C4—H4	125.8	C25—C24—H24	125.3
Ru1—C4—H4	125.8	Ru2—C24—H24	125.3
C4—C5—C1	107.8 (10)	C24—C25—C21	105.7 (11)
C4—C5—Ru1	72.1 (7)	C24—C25—Ru2	71.9 (7)
C1—C5—Ru1	71.3 (6)	C21—C25—Ru2	69.9 (6)
C4—C5—H5	126.0	C24—C25—H25	127.1
C1—C5—H5	126.0	C21—C25—H25	127.1
Ru1—C5—H5	126.0	Ru2—C25—H25	127.1
C7—C6—C10	107.8 (10)	C27—C26—C30	108.2 (10)
C7—C6—C12	124.8 (11)	C27—C26—C32	128.3 (11)
C10—C6—C12	127.4 (11)	C30—C26—C32	123.3 (10)
C7—C6—Ru1	71.0 (6)	C27—C26—Ru2	71.3 (6)
C10—C6—Ru1	71.6 (6)	C30—C26—Ru2	71.1 (6)
C12—C6—Ru1	121.8 (8)	C32—C26—Ru2	119.6 (7)
C8—C7—C6	108.2 (11)	C28—C27—C26	108.6 (11)
C8—C7—Ru1	71.5 (7)	C28—C27—Ru2	71.2 (7)
C6—C7—Ru1	70.4 (7)	C26—C27—Ru2	70.7 (6)
C8—C7—H7	125.9	C28—C27—H27	125.7
C6—C7—H7	125.9	C26—C27—H27	125.7
Ru1—C7—H7	125.9	Ru2—C27—H27	125.7
C7—C8—C9	107.0 (11)	C27—C28—C29	107.3 (11)
C7—C8—Ru1	70.4 (7)	C27—C28—Ru2	70.9 (7)
C9—C8—Ru1	71.2 (7)	C29—C28—Ru2	71.1 (7)
C7—C8—H8	126.4	C27—C28—H28	126.3
C9—C8—H8	126.4	C29—C28—H28	126.3
Ru1—C8—H8	126.4	Ru2—C28—H28	126.3
C10—C9—C8	108.4 (11)	C30—C29—C28	109.4 (11)
C10—C9—Ru1	70.8 (7)	C30—C29—Ru2	71.2 (7)
C8—C9—Ru1	70.1 (7)	C28—C29—Ru2	70.5 (7)
C10—C9—H9	125.8	C30—C29—H29	125.3
C8—C9—H9	125.8	C28—C29—H29	125.3
Ru1—C9—H9	125.8	Ru2—C29—H29	125.3
C9—C10—C6	108.6 (11)	C29—C30—C26	106.5 (10)
C9—C10—Ru1	72.1 (7)	C29—C30—Ru2	71.3 (6)
C6—C10—Ru1	70.0 (6)	C26—C30—Ru2	69.8 (6)
C9—C10—H10	125.7	C29—C30—H30	126.7
C6—C10—H10	125.7	C26—C30—H30	126.7
Ru1—C10—H10	125.7	Ru2—C30—H30	126.7
O1—C11—C1	125.0 (13)	O21—C31—C21	123.5 (13)
O1—C11—H11	117.5	O21—C31—H31	118.3
C1—C11—H11	117.5	C21—C31—H31	118.3
O2—C12—C6	125.7 (13)	O22—C32—C26	125.1 (13)
O2—C12—H12	117.1	O22—C32—H32	117.4
C6—C12—H12	117.1	C26—C32—H32	117.4
			
C5—C1—C2—C3	1.4 (12)	C25—C21—C22—C23	0.8 (13)
C11—C1—C2—C3	175.9 (10)	C31—C21—C22—C23	176.9 (10)
Ru1—C1—C2—C3	63.0 (8)	Ru2—C21—C22—C23	62.7 (8)
C5—C1—C2—Ru1	−61.6 (7)	C25—C21—C22—Ru2	−61.9 (8)
C11—C1—C2—Ru1	112.9 (10)	C31—C21—C22—Ru2	114.2 (11)
C1—C2—C3—C4	−0.6 (13)	C21—C22—C23—C24	0.5 (14)
Ru1—C2—C3—C4	61.3 (8)	Ru2—C22—C23—C24	62.4 (9)
C1—C2—C3—Ru1	−61.9 (8)	C21—C22—C23—Ru2	−61.9 (7)
C2—C3—C4—C5	−0.4 (14)	C22—C23—C24—C25	−1.6 (15)
Ru1—C3—C4—C5	60.8 (8)	Ru2—C23—C24—C25	59.9 (9)
C2—C3—C4—Ru1	−61.2 (8)	C22—C23—C24—Ru2	−61.5 (8)
C3—C4—C5—C1	1.3 (13)	C23—C24—C25—C21	2.0 (14)
Ru1—C4—C5—C1	62.8 (7)	Ru2—C24—C25—C21	62.1 (7)
C3—C4—C5—Ru1	−61.5 (8)	C23—C24—C25—Ru2	−60.1 (9)
C2—C1—C5—C4	−1.6 (12)	C22—C21—C25—C24	−1.7 (12)
C11—C1—C5—C4	−175.9 (10)	C31—C21—C25—C24	−178.0 (10)
Ru1—C1—C5—C4	−63.3 (8)	Ru2—C21—C25—C24	−63.5 (8)
C2—C1—C5—Ru1	61.7 (7)	C22—C21—C25—Ru2	61.8 (8)
C11—C1—C5—Ru1	−112.6 (11)	C31—C21—C25—Ru2	−114.5 (11)
C10—C6—C7—C8	0.7 (14)	C30—C26—C27—C28	0.6 (12)
C12—C6—C7—C8	−177.8 (11)	C32—C26—C27—C28	−174.8 (10)
Ru1—C6—C7—C8	−61.8 (9)	Ru2—C26—C27—C28	−61.4 (8)
C10—C6—C7—Ru1	62.5 (8)	C30—C26—C27—Ru2	61.9 (7)
C12—C6—C7—Ru1	−116.0 (11)	C32—C26—C27—Ru2	−113.4 (11)
C6—C7—C8—C9	−1.0 (14)	C26—C27—C28—C29	−1.1 (13)
Ru1—C7—C8—C9	−62.2 (9)	Ru2—C27—C28—C29	−62.2 (8)
C6—C7—C8—Ru1	61.1 (8)	C26—C27—C28—Ru2	61.1 (8)
C7—C8—C9—C10	1.0 (15)	C27—C28—C29—C30	1.3 (14)
Ru1—C8—C9—C10	−60.7 (9)	Ru2—C28—C29—C30	−60.8 (8)
C7—C8—C9—Ru1	61.7 (9)	C27—C28—C29—Ru2	62.1 (8)
C8—C9—C10—C6	−0.5 (15)	C28—C29—C30—C26	−0.9 (13)
Ru1—C9—C10—C6	−60.8 (8)	Ru2—C29—C30—C26	−61.3 (7)
C8—C9—C10—Ru1	60.2 (9)	C28—C29—C30—Ru2	60.4 (9)
C7—C6—C10—C9	−0.1 (14)	C27—C26—C30—C29	0.2 (12)
C12—C6—C10—C9	178.4 (11)	C32—C26—C30—C29	175.9 (10)
Ru1—C6—C10—C9	62.1 (9)	Ru2—C26—C30—C29	62.3 (8)
C7—C6—C10—Ru1	−62.2 (8)	C27—C26—C30—Ru2	−62.1 (8)
C12—C6—C10—Ru1	116.3 (12)	C32—C26—C30—Ru2	113.6 (9)
C5—C1—C11—O1	−12.4 (18)	C22—C21—C31—O21	6.2 (19)
C2—C1—C11—O1	174.3 (12)	C25—C21—C31—O21	−178.2 (12)
Ru1—C1—C11—O1	−99.6 (13)	Ru2—C21—C31—O21	94.9 (14)
C7—C6—C12—O2	−178.4 (13)	C27—C26—C32—O22	−6.7 (19)
C10—C6—C12—O2	3 (2)	C30—C26—C32—O22	178.6 (12)
Ru1—C6—C12—O2	93.6 (15)	Ru2—C26—C32—O22	−95.5 (13)

### Hydrogen-bond geometry (Å, º)

Cg is the centroid of the C1–C5 ring.

**Table:**

*D*—H···*A*	*D*—H	H···*A*	*D*···*A*	*D*—H···*A*
C2—H2···O21	0.98	2.64	3.456 (16)	141
C3—H3···O1^i^	0.98	2.62	3.536 (15)	156
C4—H4···O21^i^	0.98	2.45	3.406 (16)	164
C9—H9···O22^i^	0.98	2.42	3.370 (16)	162
C23—H23···O1^ii^	0.98	2.44	3.379 (17)	161
C28—H28···O2^ii^	0.98	2.43	3.392 (16)	167
C30—H30···O2^iii^	0.98	2.63	3.436 (14)	140
C8—H8···*Cg*^i^	0.98	2.78	3.546 (15)	135

Symmetry codes: (i) −*x*+2, *y*−1/2, −*z*+1/2; (ii) −*x*+1, *y*−1/2, −*z*+1/2; (iii) −*x*+3/2, −*y*+1, *z*+1/2.

## References

[bb1] Balavoine, G. G. A., Doisneau, G. & Fillebeen-Khan, T. (1991). *J. Organomet. Chem.* **412**, 381–382.

[bb2] Braga, D. & Grepioni, F. (1997). *Acc. Chem. Res.* **30**, 81–87.

[bb3] Braga, D., Paganelli, F., Tagliavini, E., Casolari, S., Cojazzi, G. & Grepioni, F. (1999). *Organometallics*, **18**, 4191–4196.

[bb4] Bruker (2012). *APEX2*, *SAINT* and *SADABS*. Bruker AXS Inc., Madison, Wisconsin, USA.

[bb5] Dai, L.-X. & Hou, X.-L. (2010). Editors. *Ferrocenes in Asymmetric Catalysis: Synthesis and Applications*, ch. 7–8. Weinheim: Wiley VCH.

[bb6] Desiraju, G. R. (1996). *Acc. Chem. Res.* **29**, 441–449. 10.1021/ar950135n 23618410

[bb7] MacGillivray, L. R., Spinney, H. A., Reid, J. L. & Ripmeester, J. A. (1999). *J. Chem. Crystallogr.* **29**, 865–869.

[bb8] Macrae, C. F., Bruno, I. J., Chisholm, J. A., Edgington, P. R., McCabe, P., Pidcock, E., Rodriguez-Monge, L., Taylor, R., van de Streek, J. & Wood, P. A. (2008). *J. Appl. Cryst.* **41**, 466–470.

[bb9] Mueller-Westerhoff, U. T., Zheng, Y. & Ingram, G. (1993). *J. Organomet. Chem.* **463**, 163–167.

[bb10] Muratov, D. V., Romanov, A. S. & Kudinov, A. R. (2014). *Russ. Chem. Bull.* **63**, 2485–2492.

[bb11] Nesmeyanov, A. N., Lubovich, A. A. & Gubin, S. P. (1972). *Russ. Chem. Bull.* **21**, 1761–1764.

[bb12] Rausch, M. D., Fischer, E. O. & Grubert, H. (1960). *J. Am. Chem. Soc.* **82**, 76–82.

[bb13] Sanders, R. & Mueller-Westerhoff, U. T. (1996). *J. Organomet. Chem.* **512**, 219–224.

[bb14] Sheldrick, G. M. (2008). *Acta Cryst.* A**64**, 112–122. 10.1107/S0108767307043930 18156677

[bb15] Sheldrick, G. M. (2015). *Acta Cryst.* C**71**, 3–8.

[bb16] Štěpnička, P. (2008). Editor. *Ferrocenes: Ligands, Materials and Biomolecules*, ch. 8–12, 13. Chichester: J. Wiley & Sons.

[bb17] Trotter, J. (1963). *Acta Cryst.* **16**, 571–572.

[bb30] Wavefunction (2017). *Spartan ’16*. Version 2.0.1. Wavefunction Inc., Irvine, California, USA.

